# Integrated gut microbiome and metabolome analyses identified fecal biomarkers for bowel movement regulation by *Bifidobacterium longum* BB536 supplementation: A RCT

**DOI:** 10.1016/j.csbj.2022.10.026

**Published:** 2022-10-25

**Authors:** Yuya Nakamura, Shinya Suzuki, Shinnosuke Murakami, Yuichiro Nishimoto, Koichi Higashi, Naoki Watarai, Junpei Umetsu, Chiharu Ishii, Yutaro Ito, Yuka Mori, Mamiko Kohno, Takuji Yamada, Shinji Fukuda

**Affiliations:** a Metagen Inc., 246-2 Kakuganji, Tsuruoka, Yamagata 997-0052, Japan; b Department of Life Science and Technology, Tokyo Institute of Technology, 2-12-1 Ookayama, Meguro-ku, Tokyo 152-8550, Japan; c Education Academy of Computational Life Science (ACLS), 2-12-1 Ookayama, Meguro-ku, Tokyo 152-8550, Japan; d Institute for Advanced Biosciences, Keio University, 246-2 Kakuganji, Tsuruoka, Yamagata 997-0052, Japan; e National Institute of Genetics, Genome Evolution Laboratory, Yata 1111, Mishima 411-8540, Japan; f MORISHITA JINTAN CO., LTD, Health Care Product Department, Research & Development Division, 1-2-40 Tamatsukuri, Chuo-ku, Osaka 540-8566, Japan; g Transborder Medical Research Center, University of Tsukuba, 1-1-1 Tennodai, Tsukuba, Ibaraki 305-8575, Japan; h Gut Environmental Design Group, Kanagawa Institute of Industrial Science and Technology, 3-25-13 Tonomachi, Kawasaki-ku, Kawasaki, Kanagawa 210-0821, Japan; i Laboratory for Regenerative Microbiology, Juntendo University Graduate School of Medicine, Hongo, Tokyo 113-8421, Japan

**Keywords:** AUROC, area under the receiver operating characteristic curve, SCFAs, short-chain fatty acids, CE-TOFMS, capillary electrophoresis time-of-flight mass spectrometry, CSA, D-camphor-10-sulfonic acid, ESVs, exact sequence variants, FDR, false discovery rate, IBD, inflammatory bowel disease, IBS, irritable bowel syndrome, ITT, intention-to-treat, MCMC, Markov Chain Monte Carlo, MDS, multidimensional scaling, NRs, nonresponders, PP, per-protocol population, PSRF, potential scale reduction factor, SRs, strong responders, WAIC, Widely Applicable Information Criterion, WRs, weak responders, Bifidobacteria, Probiotics, Gut microbiota, 16S rRNA gene sequence, Metabologenomics, Machine learning

## Abstract

**Background:**

*Bifidobacterium longum* BB536 supplementation can be used to regulate bowel movements in various people, including healthy subjects and patients with irritable bowel syndrome (IBS); however, individuals vary in their responses to *B. longum* BB536 treatment. One putative factor is the gut microbiota; recent studies have reported that the gut microbiota mediates the effects of diet or drugs on the host. Here, we investigated intestinal features, such as the microbiome and metabolome, related to *B. longum* BB536 effectiveness in increasing bowel movement frequency.

**Results:**

A randomized, double-blind controlled crossover trial was conducted with 24 adults who mainly tended to be constipated. The subjects received a two-week dietary intervention consisting of *B. longum* BB536 in acid-resistant seamless capsules or similarly encapsulated starch powder as the placebo control. Bowel movement frequency was recorded daily, and fecal samples were collected at several time points, and analyzed by metabologenomic approach that consists of an integrated analysis of metabolome data obtained using mass spectrometry and microbiome data obtained using high-throughput sequencing. There were differences among subjects in *B. longum* intake-induced bowel movement frequency. The responders were predicted by machine learning based on the microbiome and metabolome features of the fecal samples collected before *B. longum* intake. The abundances of eight bacterial genera were significantly different between responders and nonresponders.

**Conclusions:**

Intestinal microbiome and metabolome profiles might be utilized as potential markers of improved bowel movement after *B. longum* BB536 supplementation. These findings have implications for the development of personalized probiotic treatments.

## Graphical abstract

**Figure:**
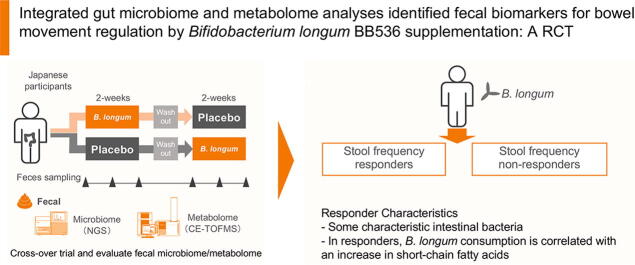

## 1 Introduction

Constipation is a globally common gastrointestinal disorder affecting people of all ages. Constipation has been reported in 5.4 % to 26.2 % of the general population [1], [2], [3], and constipation is known to occur in patients of many disorders, including irritable bowel syndrome (IBS), Parkinson's disease, and kidney disease [4], [5], [6]. Several diets are used to relieve constipation. For example, dietary fiber and magnesium have been reported to increase bowel movement frequency [3], [7], [8], [9]. In addition, in recent years, fermented products such as yogurt have attracted attention because they are useful in relieving constipation. *Bifidobacterium* is a representative microbial probiotic genus that is frequently used to regulate bowel movements [10]. Multiple randomized controlled trials have shown that bifidobacteria intake increases bowel movement frequency [11], [12], [13], [14] or shortens the gut transit time [15], [16]. However, the response to bifidobacteria supplementation varies from subject to subject. Some trials have reported that bifidobacteria supplementation alleviates IBS symptoms, while others have not observed an effect on the treatment of IBS [17], [18].

One factor that might account for these different observations is the composition of the gut microbiota. Indeed, individual differences in gut microbiota composition are known to affect probiotic-mediated stimulation [19]. Recent studies based on high-throughput sequencing of the human microbiome have elucidated how the host response to diet is affected by intestinal microbes [20], [21], [22], [23], [24], [25]. For example, gut microbial dysbiosis induced by the excess consumption of artificial sweeteners impairs the host's glucose tolerance metabolism [20], and excess consumption of dietary emulsifiers promotes colitis and metabolic syndrome via the gut microbiome [21]. In addition, multi-species probiotics capsules were administered to women with IBS-D in another study in which patients with beneficial changes in inflammatory markers had higher baseline proportions of *Faecalibacterium*, *Leuconostoc*, and *Odoribacter* compared to corresponding non-responders [26]. When *Bifidobacterium longum* AH1206 was orally administered to humans, there were individual differences in whether bacteria were colonized, and a metabolic niche was necessary for stable persistence [27].

These individual differences in the response can be predicted with some accuracy from omics data for the gut microbiome [22], [23]. In patients with inflammatory bowel disease (IBD), which is one of the most well-known gastrointestinal disorders, the response to anti-integrin biologic therapy is predictable from gut microbial function [28]. With the prediction of the effects of drugs or a diet based on the state of the intestinal environment, personalized prescriptions might be possible. In other words, the companion diagnosis based on the individual’s intestinal environment might enable personalized health care and reduce medical costs.

**Fig. 1:**
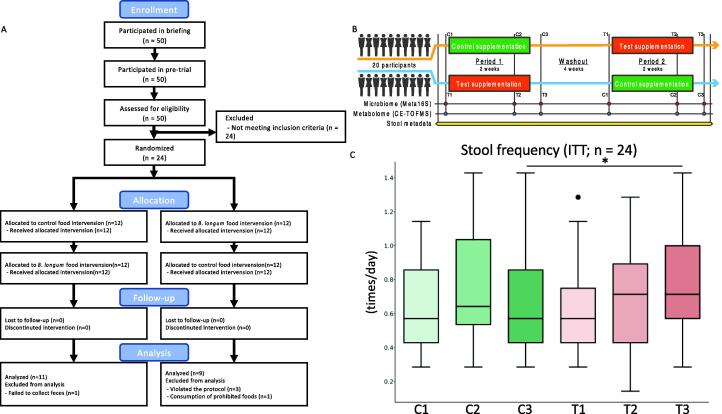
Schematic of the randomized controlled trial for the influence of bifidobacteria intake on bowel movement. A: Flow diagram of the phases of the randomized double-blind placebo-controlled crossover study. B: The illustration indicates the design of the randomized double-blind controlled trial to investigate the increase in bowel movement frequency due to the intake of *B. longum* BB536 encapsulated in an acid-resistant seamless capsule. C: The boxplots indicate the bowel movement frequency per day. *, *p* < 0.05, Wilcoxon signed-rank test. ITT: Intention-To-Treat.

In this study, we focused on *B. longum* BB536, commercially available probiotic product in Japan, and conducted a randomized double-blind controlled crossover trial in adults with constipation to determine the effect of *B. longum* BB536 supplementation on bowel movement frequency (Fig. 1A, B). In addition, we conducted 16S rRNA gene-based microbiome and metabolome analyses of fecal samples collected during the trial to evaluate the effect of *B. longum* BB536 supplementation on the intestinal environment. Furthermore, we also focused on the individual differences in the response, and the effect of *B. longum* BB536 intake on increasing the bowel movement frequency for each individual was estimated using a Bayesian statistical model.

## 2 Results

### 2.1 The effect of *B. longum* BB536 intake on bowel movement frequency

In this study, we conducted a randomized double-blind controlled crossover trial to quantify the increase in bowel movement frequency in response to the intake of acid-resistant seamless capsules containing *B. longum* BB536. All 24 subjects completed the trial. We analyzed the microbiome and metabolome profiles of fecal samples collected during the trial period. Four out of 24 subjects were not analyzed for microbiome and metabolome profiles because they violated the protocols. We confirmed that the bowel movement frequency increased during the test intervention period (Fig. 1C, Table S1). As a result, the bowel movement frequency increased compared to the control group in the second week of the intervention period (intention-to-treat (ITT) population: *p* = 0.0334; per-protocol (PP) population: *p* = 0.0492). The results for other stool metadata (amount, shape and color) are listed in Table S2.

### 2.2 The intestinal environment of each individual was stable for the *B. longum* BB536 supplement intervention

**Fig. 2:**
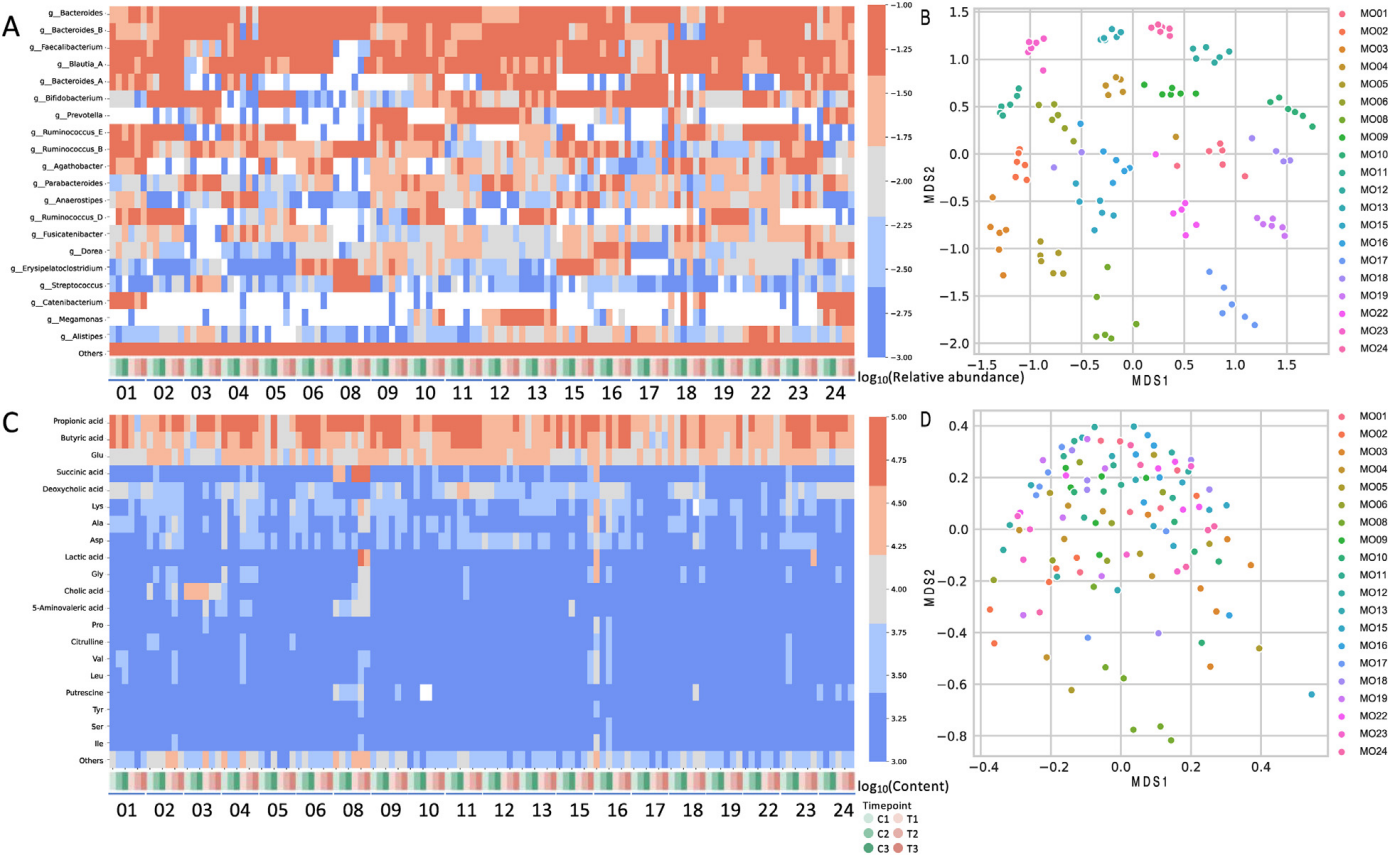
Overall view of the metabologenomic data. A: The heatmap represents the relative abundance of the top 20 genera. If the value is less than 0.001, it is represented in white. B: The scatter plot shows the results of MDS using beta diversity (Spearman correlation coefficient distance) calculated from the microbiome composition. C: The heatmap represents the abundance of the top 20 metabolites in the fecal sample. If the value is less than 1000, it is represented in white. D: A scatter plot showing the results of MDS using beta diversity (Spearman correlation coefficient distance) calculated from the metabolite composition. The same color was used for each subject.

The 16S rRNA gene region was amplified based on the DNA extracted from the fecal samples and sequenced using a high-throughput sequencer to determine how the microbiome composition of the subjects changed over the course of the experiment. Based on these sequencing data, the relative abundance, alpha diversity, and beta diversity at the bacterial genus level were calculated. Furthermore, metabolites were extracted from the fecal samples and analyzed by coupling capillary electrophoresis with electrospray ionization time-of-flight mass spectrometry (CE-TOFMS). Overall, 322 metabolites were detected. Alpha diversity representing the intra-sample complexity of the microbiome composition and beta diversity representing the inter-sample diversity were calculated from the metabolome data. No overall trends were observed in intestinal microbiome and metabolome composition in either of the two intervention groups (Fig. 2A, C). The results of multidimensional scaling (MDS) analysis using beta diversity to analyze similarity between all the samples showed that the microbiome and metabolome composition of the same subject were similar over the course of the experiment (Fig. 2B, D, Fig. S1A, B). These results suggested that the effects of individual differences in the intestinal microbiome and metabolome compositions were greater than the effect of the intervention.

Subsequently, differences in individual gut microbes and metabolites were analyzed. Generally, crossover studies are considered to possibly have a carry-over effect. In this study, Spearman’s distances for the intestinal microbiome and metabolome were compared between group A and group B, but no carry-over effect was observed (Fig. S2). Next, the Wilcoxon signed-rank test (two-sided test) was performed to compare the difference in gut microbes and metabolites between the control and test intervention groups at same time point (*C*2-T2 and C3-T3). The results showed that some of the bacterial genera were different in the test intervention group from those in the other groups (*p* < 0.05, not corrected). However, when false discovery rate (FDR) correction was applied, no changes were detected (Table S3), and no significant change was detected in alpha diversity (Table S4, Fig. S3). Overall, our results indicate that the effect of *B. longum* BB536 intake on the intestinal microbiome and metabolome was small relative to the effect of individual differences in the intestinal environment, and the intestinal environment of each individual was robust for the *B. longum* BB536 supplement intervention.

### 2.3 Determination of the bowel responders using a Bayesian Weibull regression analysis

The extent of the increase in bowel movement frequency due to *B. longum* supplementation or a placebo effect varied among subjects. To test whether the microbiome or metabolome features of an individual’s intestinal environment varied with the magnitude of response, we first quantified the magnitude of the treatment effect in each individual using a Bayesian statistical model. The models were formulated to precisely estimate the sizes of the placebo effect and the test supplement effect. In the models, the bowel movement interval of each individual was used based on the tendency of bowel movement to occur with the lapse of time. The bowel movement intervals were calculated from the daily frequency of bowel movements. The parameters for the models were estimated by Bayesian statistical fitting of the bowel movement interval to the models. Subject MO03 was excluded from further analysis because this subject's bowel movements consistently occurred once a day throughout the whole observation period (85 days).

We prepared several models to investigate individual differences, some of which assumed a constant treatment effect across individuals and some variation among individuals (Table S5). The models were evaluated using the Widely Applicable Information Criterion (WAIC) [29]. The lower the WAIC indicates that the model explained the data without overfitting. The WAIC is an index of prediction performance for unknown data. Among the models, the model considering the individual placebo effect and test supplement effect had the lowest WAIC (Table S6). These results indicated that some subjects had increasing bowel movement frequencies as a result of intake of *B. longum* BB536 supplement; we defined these subjects as “bowel responders”.

**Fig. 3:**
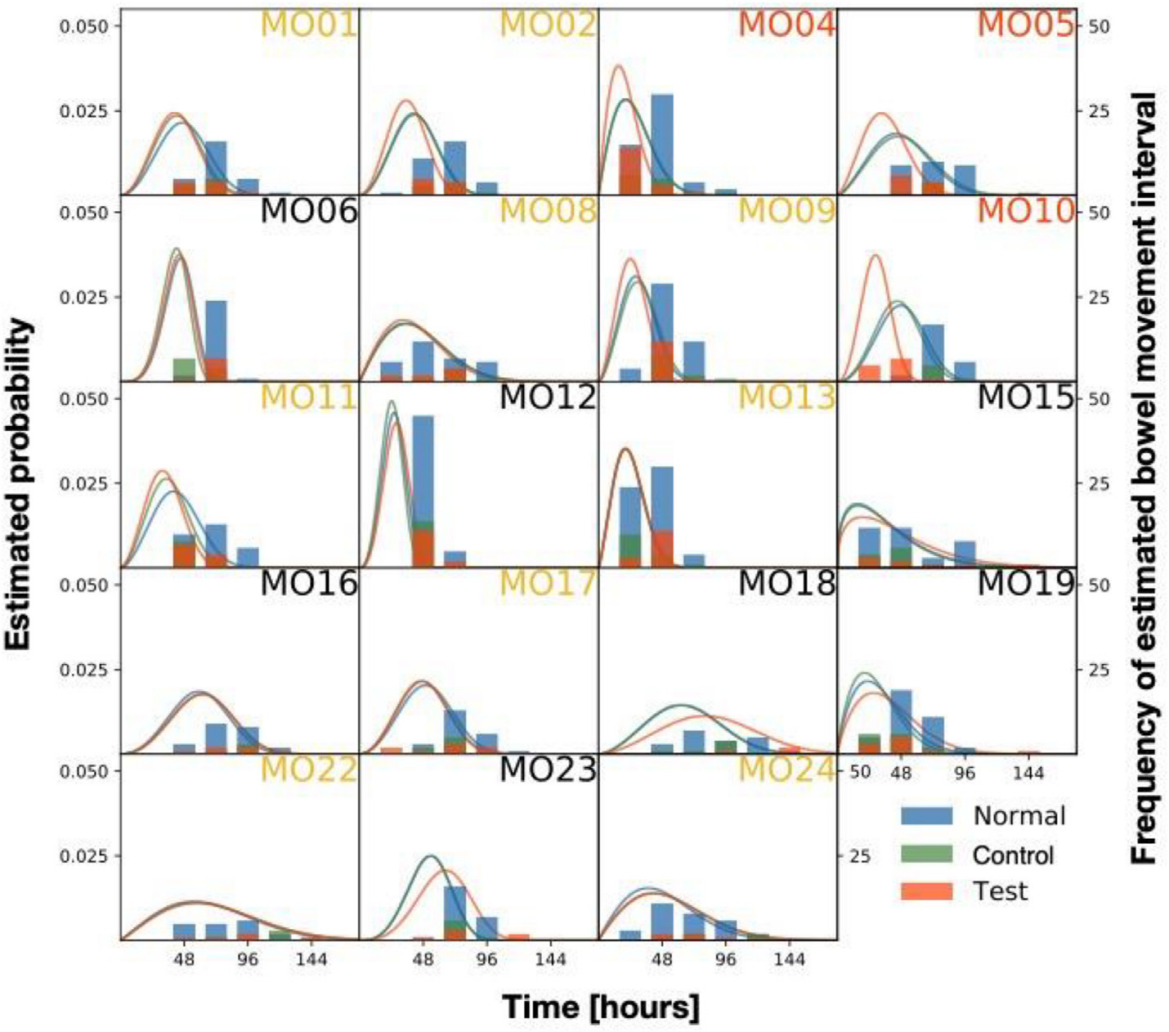
Histogram of the bowel movement time interval and probability density function of the Weibull distribution. The vertical axis on the left shows the value of the probability density function, and the vertical axis on the right shows the frequency of the estimated bowel movement interval in the whole observation period (85 days). The responder status of the subject was defined based on the Bayesian posterior distribution: SRs are indicated with orange labels, WRs are indicated with yellow labels, and NRs are indicated with black labels. (For interpretation of the references to color in this figure legend, the reader is referred to the web version of this article.)

Using the model with the best WAIC, the estimated posterior mean of each parameter and calculated its Bayesian credible intervals for each individual (Table S6, Fig. 3). The effects of the test supplement in subjects MO04, MO05, and MO10 were strong, as evidenced by the lower limit of 95 % Bayesian credible intervals of the predicted distribution greater than zero. Therefore, MO04, MO05, and MO10 were defined as strong responders (SRs). In addition, there were 9 other subjects with estimated posterior mean values greater than zero, who were defined as weak responders (WRs). The remaining subjects were defined as nonresponders (NRs).

### 2.4 Machine learning enabled us to predict bowel responders using metabologenomic data obtained before *B. longum* BB536 intake

**Fig. 4:**
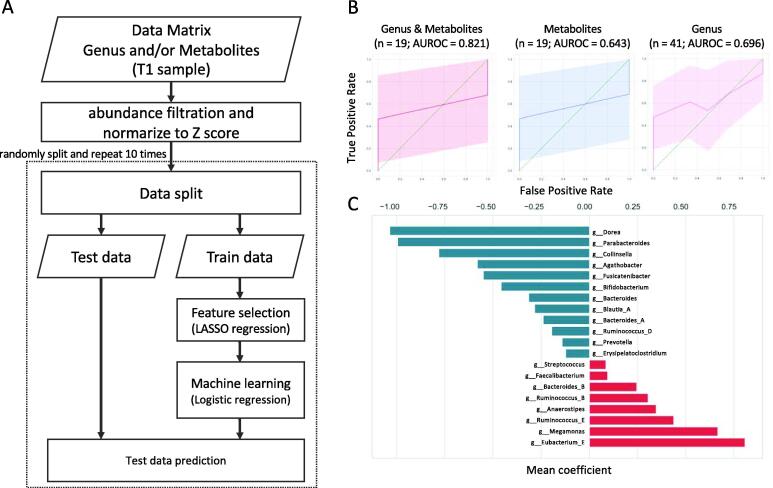
Machine learning-based responder prediction using intestinal environmental features identified before the intake of the test supplement. A: A flow chart of the machine learning algorithm for responder prediction. Feature selection by LASSO regression and machine learning by logistic regression algorithm were used. B: A receiver operating characteristic (ROC) curve when predicting, from intestinal omics data collected before intake of the test supplement, who will be a responder (SR and WR) (i.e., experience a significant increase in bowel movement frequency). C: Regression coefficient of the logistic regression model with respect to the responder score for the parameter with the highest accuracy in the 7-fold stratified validation using bacterial genera and data collected before the test supplement intake began.

In parallel with our experimental observations of the intestinal environmental factors that determine the response to supplementation with *B. longum* BB536, we also investigated whether responders could be predicted from intestinal environmental features before *B. longum* BB536 intake by a machine learning approach. We used the machine learning method combined with LASSO regression and logistic regression and predicted whether a subject would be a responder from the metabologenomic data (fecal microbiome and metabolome data) before the test intervention to assess this strategy (Fig. 4A). The composition of bacterial genera, metabolites, or their combinations were used as explanatory variables for predicting responders, and we estimated parameters and evaluated the performance of machine learning using a grid search and stratified cross-validation. For the stratified cross-validation, we chose 7-fold stratified cross-validation to use additional training data, considering that the 7 subjects were NRs. As a result, all responders (SRs and WRs) were predicted, and the highest performance (area under the receiver operating characteristic curve (AUROC) = 0.821) was observed when using metabolite and bacterial genus data (Fig. 4B, Table 1). Although high performances were observed, the training data only include 19 samples, which may have caused overtraining of the model. We expanded the sample size by adding data from a 2nd cohort in the study to validate the prediction model (n = 22; only microbiome data). First, we conducted the responder determination, and although no SRs were identified, 11 WRs were determined (Table S6). Although the prediction model using data from the 1st cohort was not able to detect the responders in the 2nd cohort (AUROC = 0.500), the prediction model learned from combined data from the 1st and 2nd cohorts and was able to predict responders with high performance (AUROC = 0.696). The logistic regression analysis using the bacterial genus data with a parameter set for the highest AUROC showed that the *g__Dorea*, *g__Parabacteroides, g__Collinsella*, *g__Agathobacter*, *g__Fusicatenibacter*, *g__Bifidobacterium*, *g__Bacteroides*, *g__Blautia_A*, *g__Bacteroides_A*, *g__Ruminococcus_D*, *g__Prevotella*, *g__Erysipelatoclostridium*, *g__Streptococcus*, *g__Faecalibacterium*, *g__Bacteroides_B*, *g__Ruminococcus_B*, *g__Anaerostipes*, *g__Ruminococcus_E*, *g__Megamonas*, *and g__Eubacterium_E* levels were involved in predicting responders (Fig. 4C). Although the exact mechanisms underlying the *B. longum*-induced effects of the gut microbiota on bowel movement frequency have yet to be elucidated, our machine learning approach allowed us to predict the patients who would respond to *B. longum* BB536 intake with an increased bowel movement frequency.

**Table 1:** Summary of the responder predictions using machine learning.

Data	Microbiome relative abundance threshold**	Metabolome content abundance threshold**	LASSO regression parameter alpha**	Average AUROC*	Accuracy	F-measure
Genus and metabolite (n = 19)	0	100	0.001	0.821	0.760	0.832
Metabolite (n = 19)	–	100	0.0001	0.643	0.550	0.563
Genus (n = 19)	0	–	0.001	0.793	0.773	0.841
Genus (n = 41)	0.01	–	0.001	0.696	0.649	0.673

* AUROC; area under the receiver operating characteristic curve.

** For each data set, the parameters, AUROC, accuracy and F-measure at the highest performance are shown.

### 2.5 Differences in the intestinal environment between bowel responders and nonresponders, as determined using a Bayesian statistical model

**Fig. 5:**
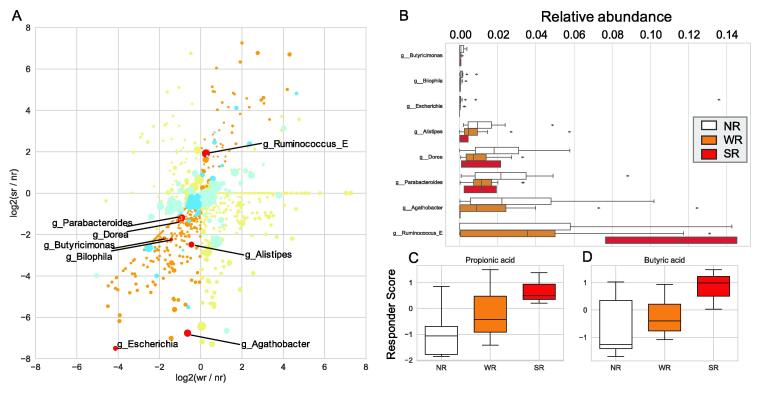
Intestinal features related to the increasing bowel movement frequency due to *B. longum* BB536 intake. A: A scatter plot showing the fold change in microbiota and metabolite abundances in responders compared with NRs. The y-axis represents the log 2 value of the SR compared with the NR fold change (FCs), and the x-axis represents the log 2 value of the WR compared with the NR fold change (FCw). The warm plot corresponds to the microbiota, and its size represents the mean relative abundance. The light orange plot indicates FCs > FCw > 0 or FCs < FCw < 0. Red represents bacterial genera that were subjected to the Jonckheere-Terpstra trend test and had a *p* value < 0.05. The cold plot corresponds to metabolites, and its size represents the mean fecal content. B: Boxplots show the baseline (T1) relative abundance of bacterial genera in NRs, WRs and SRs for which significant differences were detected using the Jonckheere-Terpstra trend test. C, D: Boxplots show changes in the levels of propionic acid (C) and butyrate (D) induced by the intervention (responder score). Values were calculated based on the values measured after 1 week of intake (T2 and C2). Significant differences were detected between NRs, WRs, and SRs using the Jonckheere-Terpstra trend test (*p* < 0.05). (For interpretation of the references to color in this figure legend, the reader is referred to the web version of this article.)

We then calculated fold changes for each bacterial genus and metabolite abundance for the SR and W*R* groups and compared them to those for the NR group (FCs and FCw, respectively). To detect features with large or small relative abundances when the effect of the *B. longum* BB536 supplement was strong, the genera and metabolites with fold change values following the order FCs > FCw > 1 or FCs < FCw < 1 were extracted, and the trends were verified using the Jonckheere-Terpstra trend test. FCs and FCw were calculated from the values at T1, the time point prior to test food intervention, from 1st and 2nd cohort data. Increasing trends were detected for one genus, and decreasing trends were detected for 7 genera (*p* < 0.05) (Fig. 5A). The relative abundance of g__*Ruminococcus*_E was significantly higher in the responder groups than in the NR group. Conversely, *g__Agathobacter*, *g__Alistipes*, *g__Bilophila*, *g__Butyricimonas*, g*__Dorea*, *g__Escherichia* and *g__Parabacteroides* were significantly less abundant in the responder groups than in the NR group (Fig. 5B; Jonckheere-Terpstra trend test, *p* < 0.05).

Next, differences in alterations in the gut microbiome induced by the intervention between NRs, SRs and WRs were evaluated. Because no gut environment data were obtained after intervention in the 2nd cohort, this alteration analysis was performed only with 1st cohort data. Some alterations in bacteria and metabolites were significantly different among NRs, WRs, and SRs (Table S7). Of these bacteria, we searched for common bacteria with the eight bacteria that differed at baseline. However, no common bacteria were identified. The abundance of propionate and butyrate, which are major metabolites produced by gut microbiota, showed trends toward a greater increase in responders (Fig. 5C, D; *p* = 0.0361 for butyrate, *p* = 0.0118 for propionate; Jonckheere-Terpstra trend test). Statistical significance of trends for propionate and butyrate levels were only observed when comparing groups at T2; however, the trends still partially existed in the comparison at T3. Taken together, these results revealed that eight genera existed with significantly different abundances in responders who had a bowel movement frequency-increasing effect due to *B. longum* BB536 intake compared with those in NRs. Furthermore, the responders showed a greater increase in propionate and butyrate abundance after *B. longum* BB536 intake.

## 3 Discussion

The bowel movement responses to probiotic intake vary among individuals [11], and the gut microbiota is a key factor that may account for these differences [19]. Thus, in the present study, we accurately quantified individual differences in the increase in bowel movement frequency in response to intake of *B. longum* BB536 contained in acid-resistant seamless capsules using a statistical model and defined the responders as those with a noticeably increased bowel movement frequency. In addition, the machine learning analysis revealed that responders could be predicted from features of the intestinal environment before the initiation of *B. longum* BB536 supplement intake, and the predictive performance was improved when using both microbiome and metabolome data. Based on these results, accurate quantification of the individual response intensity and machine learning predictions may enable companion diagnostics for the response to *B. longum* BB536 supplementation based on the intestinal environment.

Our finding showing that the use of both microbiome and metabolome data improved the predictive performance of machine learning indicates that an integrative analysis of multiomics data is important for analyzing the response that depends on the individual intestinal environment. In recent years, multiple studies have predicted responses to interventions from metagenomic datasets alone, including datasets of the taxonomic composition [24], [25], [30]; incorporating metabolome data may enhance the predictive performance. Additionally, those results may indicate the possibility that intestinal metabolism is affected by the individual differences in bowel movement frequency. Moreover, previous studies have reported that, in addition to increasing bowel movement frequency, intake of the *B. longum* BB536 strain encapsulated in an acid-resistant seamless capsule results in a change in fecal color from dark to light, a change in fecal shape from a hard or watery state to a soft state, and a decrease in the amount of ammonia in feces [14], [31]. If individual responses with these effects were accurately quantified, then metabologenomic data may be able to predict which subjects will have these responses, as well as which individuals will have an increased bowel movement frequency. In addition, several studies have suggested that individual responses to the intake of other dietary factors might be predicted from the intestinal environment [22], [23], [32]. According to these results, we expect that the effect of probiotics will be predicted from the intestinal environment data before intake in the future, and the development of personalized health care and medical businesses targeting the intestinal environment are expected using this prediction system.

We established an accurate Bayesian statistical model to estimate the effect of *B. longum* BB536 intake on increasing the bowel movement frequency, and this model revealed which subjects were responders or NRs in this study. This model enabled us to detect shortening of the bowel movement interval due to the intake of *B. longum* BB536, consistent with previous research [15], [16]. In addition, because the Bayesian estimation of the parameters enabled us to calculate a correct interval, this model achieved an accurate quantitative evaluation of the effect of *B. longum* BB536 intake on bowel movement frequency, which has been difficult to achieve using threshold-based responder estimates [23].

Few significant differences in the microbiome and metabolome were detected among the baseline, *B. longum* BB536 intake, and control supplement samples. This lack of significant differences in the intestinal environment is related to interindividual variation, which masks the effect of the intake of *B. longum* BB536. This finding has also been reported in several studies that have observed changes in the gut microbiome composition induced by probiotics or dietary interventions. Thus, this effect is because the intestinal environment of each individual is robust, and individual differences in the intestinal environment override the effect of probiotic intake.

Our observational and machine learning results indicated that the relative abundance of 20 genera was associated with whether a subject responded well to *B. longum* BB536 intake, and, interestingly, 10 of these 20 responder-related genera belonged to the class Clostridia. Clostridia is an important bacterial group that produces mainly short-chain fatty acids (SCFAs), including butyrate, in the gut environment [33]. An imbalance of SCFAs may cause IBD and IBS because of the involvement of SCFAs in the maintenance of intestinal homeostasis [34], [35], [36]. Clostridia clusters IV and XIVa in Clostridia are reported to produce butyrate, which is related to the promotion of large intestinal peristaltic movement [33]. Among the eight genera suggested as possibly involved in the bowel responders in this study, four genera of Ruminococcaceae belong to Clostridia cluster IV, and six genera of Lachnospiraceae belong to Clostridia cluster XIVa. The bacteria in Clostridia clusters IV and XIVa cross-feed with *Bifidobacterium* and metabolize the inulin-type fructans derived from *Bifidobacterium* to produce butyrate [37], [38], [39]. We therefore suggest that the balance of bacteria belonging to Clostridia clusters IV and XIVa may be related to the increase in bowel movement frequency in response to intake of *B. longum* BB536 via butyrate or other SCFAs, and that researching the cross-feeding relationship of these bacteria might lead to a in-depth understanding of this relationship.

Our results also suggest that butyrate and propionate may play roles in the response to *B. longum* BB536 intake. Compared with NRs, responders tended to show an increase in the abundance of butyrate and propionate during the test intervention period (Fig. 4). The efficiency of butyrate production during sugar metabolism in these bacteria varies among bacterial species and habitat. Previous studies have indicated that the sugar-metabolizing abilities of *Ruminococcus bromii*, *Eubacterium rectale* (*Agathobacter rectale*), *Bacteroides thetaiotaomicron*, *Bifidobacterium adolescentis*, *Anaerostipes caccae,* and *Eubacterium hallii* vary with the habitat [38], [40]. In addition, *Ruminococcus* and *Bifidobacterium* are localized in the intestinal lumen, and *Clostridium* is localized in the intestinal lumen and on the intestinal epithelial cell surface, whereas *E. rectale* preferentially colonizes the mucous layer to produce and promote the utilization of butyrate by colonic epithelial cells [33], [41]. Taken together, these findings suggest that variations in butyrate productivity among bacteria and their locations in the large intestine may influence whether a subject responds to *B. longum* BB536 intake.

In our study, the most valuable points are the double-blind placebo-controlled design of the clinical trial and use of multi-omics features to define responsiveness to the probiotic intervention; however, three limitation points should be considered. First, 16S metagenomic data were used to evaluate the gut microbiome. Since 16S metagenomic data reveal only the taxonomic composition and cannot be used for detailed functional analysis, it is necessary to conduct a future study with shotgun metagenomics to clarify the hypothesis proposed in this study. The other limitation is that the final number of subjects without baseline analysis was 20. In this study, this was compensated for by acquiring the time-series data for each individual; however, in the future, the results must be validated by a larger cohort study. Although no such large cohort has been conducted for the effect of probiotics on bowel movement frequency, the expectations and demands for such a study have risen considerably. Third, in this study, stool samples were used, which do not reflect the entire microbiota composition of the small and large intestinal tract. More detailed intestinal tract microbiome information might be obtained from the intestinal tissue using colonoscopy.

## 4 Conclusions

In summary, our results show that the gut microbiota composition impact the effectiveness of *B. longum* BB536 supplementation in increasing bowel movement frequency, and the fecal metabologenomic data enable us to predict the effect of *B. longum* BB536 supplementation before intake. These findings have interesting implications for personalized treatment of chronic constipation. In addition, the effect of the intestinal environment on the response to intervention should be considered not only for probiotics but also for all health effects, adverse effects, and side effects for all orally administered interventions. When the effect of probiotics, diets, or drugs is dependent on the intestinal environment before intake, as it is in the present study, the demand for intestinal environment alterations to enhance the effect may also increase. Furthermore, the development of personalized health care and medical businesses targeting the intestinal environment is expected to use this intervention effect quantification and responder prediction system.

## 5 Methods

### 5.1 Trial design and recruitment

In this study, we conducted a randomized double-blind controlled crossover trial with healthy subjects from September to December 2015 to quantify the increase in bowel movement frequency in response to the intake of acid-resistant seamless capsules containing *Bifidobacterium longum* BB536. In addition, we analyzed the microbiome and metabolome profiles of fecal samples collected during the trial period (Fig. 1A, Table S8, Supplementary Protocol). The human rights of the subjects who participated in this study were protected at all times, and the study observed the Helsinki Declaration and Ethical Guidelines on Epidemiological Research in Japan referring to cases concerning standards for clinical trials of drugs. This randomized controlled trial was conducted with the approval of the clinical trial ethics review committee of Chiyoda Paramedical Care Clinic and reported to UMIN Clinical Trials Registry (UMIN000018924).

The sample size was estimated based on a previous randomized controlled trial using the probiotic strain *B. longum* BB536 as the test supplement [42]. During the study period, subjects were restricted from making any major changes in their eating habits from prior to study participation. Subjects were also required to consume as few probiotics and prebiotics as possible. In this previous trial, the bowel movement frequency during the test intervention period was 9.6 ± 0.6 [/ 2 weeks], and that during the control intervention period was 8.4 ± 0.4 [/ 2 weeks]. We applied power analysis to estimate the number of subjects with a significance level of 5 %. The statistical power was 80 %, with the expectation that 20 % of subjects would drop out in this trial. From these conditions, the number of participants required for this trial was estimated to be eight.

A preliminary bowel movement test was conducted. Among 50 Japanese participants, 24 subjects who fulfilled the criteria were selected. Age, male–female ratio, and the bowel movement status were selected before the main trial based on the preliminary results. Data from four of 24 subjects were not used in the gut microbiome and metabolome analysis, as they violated the protocols. MO07 failed to collect fecal samples during the collection period. MO14, MO20 and MO21 took the medication more than one time. MO01 took probiotics once during the pre-observation period but was included in the subsequent analysis. In these twenty subjects, the average age was 47.7 ± 5.8, and the male/female ratio was 8:12. The remaining baseline demographic and clinical characteristics did not differ significantly between the control and treatment groups (Table S9).

### 5.2 Subject characteristics and exclusion criteria

This clinical study was approved by the ethics committee and then requested to CPCC Co., Ltd. All of the participants consented to participate in this study after the study was fully explained to them.

The subject inclusion criteria were as follows: 1) aged 40 to 60 years at the time of providing consent and 2) stool frequency of three to five times per week or more than seven times per week. The exclusion criteria were as follows: 1) laparotomy surgery performed within six months before the start of the study, 2) intake of antibiotics for more than one week within six months before the start of the study, 3) allergies to the test supplement, 4) significant lifestyle changes intended during the examination period, 5) susceptibility to chronic diarrhea, 6) history of significant liver dysfunction, gastric dysfunction, or cardiovascular disease, 7) suspected chronic or acute infection, 8) known or potential pregnancy or breastfeeding, 9) participation in other trials within the month before the start of the study and 10) otherwise assessed to be inappropriate for the study by the physician involved in the study. All subjects were monitored for defecation frequency for two weeks before the start of the study, and it was confirmed that the defecation frequency was either three to five times per week or seven times per week or more. Among the subjects who completed the study, those who met any of the following conditions were excluded from further analysis: 1) considered to have met one or more of the exclusion criteria, 2) an inoculation rate of the test supplement less than 80 %, 3) large variation in the diet record or lack of confirmation of no change from the record, 4) large variation in the content of the lifestyle record or lack of confirmation of no change from the record, 5) continued or repeated ingestion of medicines, foods or supplements such as stool softeners that might influence the outcomes of the trial, and 6) otherwise assessed to be inappropriate for the study.

### 5.3 Trial intervention: dietary information

In this trial, we used acid-resistant seamless capsules containing approximately 5.0 * 10^9^ *B. longum* BB536 (0.53 g per serving) as the test supplement (MORISHITA JINTAN CO., Ltd, Japan). These capsules are spherical with a diameter of approximately 2.4 mm and consist of two types of acid-resistant layers. Approximately 90 % of encapsulated *B. longum* BB536 survive for two hours in artificial gastric fluid adjusted to pH 1.2 [12]. The acid-resistant pH-dependent disintegrating membrane, which is the outermost layer of the capsule, passes through the stomach and collapses as the surrounding pH becomes neutral in the small intestine. Subsequently, the hardened fat layer, which is an intermediate layer, is dissolved by the surface-active action of bile acid, the digestive action of lipase, and physical stimulation by intestinal motility, and then the internal bacteria are released. The released bacteria have been reported to grow in the intestine [43], [44]. For the control supplement, the same capsule containing only starch derived from potatoes, which is used as diluent base and also contained in test supplement, was used (MORISHITA JINTAN CO., ltd., Japan). The energy, protein, lipid, carbohydrate, and sodium contents were adjusted to be equivalent between the test supplement and the control supplement. In addition, the appearances of the test and control supplements were processed to be indistinguishable.

### 5.4 Trial intervention: randomization and blinding

Randomization in the trial was performed by a blocked stratified randomization method. First, the 24 subjects who passed the selection criteria were assigned to two groups (group A, group B) by stratification, taking into consideration age, male–female ratio, and bowel movement status before the trial period. Subsequently, the symbol “A” or “B”, representing either the test or control supplement, respectively, was randomly assigned to each group of subjects, and a test supplement assignment table with the test supplement symbol and the subject identification code was prepared. Immediately after the test supplement was assigned to one group, the table was sealed and maintained secure until the end of the study, such that most of the investigators were blinded to the group assignments. Each subject orally took one serving of the test or control supplement per day with as much water as they desired. One kind of intervention was taken for two weeks, and then the other kind was taken for two weeks after a four-week washout period. The capsules were stored at room temperature by the subjects until intake.

### 5.5 Trial outcomes and sample collection

The primary outcomes in this study were bowel movement frequency and the features of the intestinal environment (intestinal microbiome and metabolome). The bowel movement frequency data and data on other activities were acquired from a questionnaire that was completed once a day (Table S10). The questionnaire included life status, diet, and bowel movement status. All subjects were prohibited from consuming 1) beverages or foods containing lactic acid bacteria or bifidobacteria in large amounts, 2) beverages or foods rich in dietary fiber or oligosaccharides, 3) dietary supplements, 4) functional yoghurt, and 5) fermented soybeans (natto), and each participant noted on the questionnaire if any of these items had been consumed. The features of the intestinal environment were obtained from the stool specimen collected one week before the intervention, and after one and two weeks of the intervention. Feces were collected by each subject at home using a collection sheet (Nagaseru; OZAX, Japan) laid in the toilet bowl and a feces collection tube (feces container 54 × 28 mm; SARSTEDT AG & Co, Germany). After collection, the stool samples were stored immediately in the household freezer and then collected later and transported to the laboratory by refrigerated transport.

### 5.6 DNA extraction and 16S rRNA gene analysis

We used the protocol described in a previous report [45] for DNA extraction from stool samples. From the extracted DNA samples, the 16S rRNA gene region was amplified using the universal primers 27Fmod and 338R for the V1-V2 region of the bacterial 16S rRNA gene [46] (Fig. S4). For sequencing the amplicon DNA, a MiSeq platform (Illumina, USA) was used in paired-end mode with 600 cycles. The obtained 16S rRNA gene sequence data are available in the DDBJ DRA (DRA accession number: DRA006874).

For the 16S rRNA gene analysis, the sequenced forward and reverse reads of each sample (average number of reads: 44,441 ± 5,959) were merged using vsearch version 1.9.3 (options: --fastq_maxee 9.0 --fastq_truncqual 7 --fastq_maxdiffs 300 --fastq_maxmergelen 330 --fastq_minmergelen 280) [47]. Subsequently, we obtained high-quality reads by quality filtering using fastp with the default parameters [48]. High-quality read FASTQ files were converted to FASTA format using SeqKit [49]. The final set of high-quality reads was used to identify exact sequence variants (ESVs) using the Deblur pipeline [50]. In the pipeline, artifact sequences including PhiX were removed, and de novo chimera sequences were analyzed and removed. All ESVs were aligned to the Genome Taxonomy Database version 86 [51] using BLAST version 2.8.1 [52], where 94 % identity was used for the genus level [53]. The correspondence table of ESVs and phylogenetic information and the number of reads in each step are shown in Tables S11 and S12.

### 5.7 Metabolite extraction and analysis

The metabolome analysis was conducted as described previously, with some modifications [45], [54]. To extract metabolites from feces, samples were lyophilized using a VD-800R lyophilizer (TAITEC) for at least 24 h. Freeze-dried feces were disrupted with 3.0-mm zirconia beads by vigorous shaking (1,500 rpm for 10 min) using a Shake Master NEO homogenizer (Biomedical Science). Five hundred microliters of methanol containing the internal standards (20 μM each of methionine sulfone and d-camphor-10-sulfonic acid) were added to 10 mg of disrupted feces. Samples were further disrupted with 0.1 mm zirconia/silica beads by vigorous shaking (1,500 rpm for 5 min) using the Shake Master neo. Next, 200 μl of ultrapure water and 500 μl of chloroform were added before centrifuging at 4,600 × g for 15 min at 20 °C. Subsequently, 150 μl of the aqueous layer was transferred to a centrifugal filter tube (UltrafreeMC-PLHCC 250/pk for Metabolome Analysis, Human Metabolome Technologies) to remove protein and lipid molecules. The filtrate was centrifugally concentrated and dissolved in 50 μl of ultrapure water immediately before CE-TOFMS analysis. The measurement of extracted metabolites in both positive and negative modes was performed by CE-TOFMS. All CE-TOFMS experiments were performed using the Agilent CE capillary electrophoresis system (Agilent Technologies). Annotation tables were produced from measurements of standard compounds and were aligned with the datasets according to similar values and normalized migration times. Then, peak areas were normalized to those of the internal standards, which were methionine sulfone and d-camphor-10-sulfonic acid for cationic and anionic metabolites, respectively. Concentrations of each metabolite were calculated based on the relative peak areas and the concentrations of standard compounds. The obtained metabolome data are presented in Tables S13 and 14.

### 5.8 Bioinformatics and statistical analyses

All statistical analyses were performed using Python (version 3.7.3) and R (version 3.6.1). The Shannon diversity index was used for alpha diversity, and the Spearman correlation distance was used for beta diversity. MDS was calculated using beta diversity. A two-sided Wilcoxon signed-rank test was used to compare feces metadata (bowel movement frequency, shape, and color), microbiome data and metabolome data between the two groups (scipy version 1.3.1). The Benjamini-Hochberg false discovery rate correction (FDR) method was used for multiple test correction (statsmodels version 0.10.1). For the gut microbiome analysis, taxonomic composition data were used at the genus and ESV levels. Of these, ESV-level data were used only to calculate the alpha diversity. The genus-level data were used for the other statistical analyses. For the gut metabolome analysis, the relative area compared with that of an internal standard and content of metabolite data was used. The metabolite contents were used for prediction and feature analyses of bowel responders. In the statistical analysis of bowel responder features, a two-sided Jonckheere-Terpstra trend test was applied to NR, WR, and SR groups, and those with *p*-values less than 0.05 were extracted (clinfun version 1.0.15).

### 5.9 Bowel responder definition based on the Bayesian Weibull regression model

Using the data from the questionnaires recorded by each subject over the course of the study, subjects who strongly responded to treatment, i.e., who showed an increased bowel movement frequency, were estimated using a statistical model. An increasing effect of the test supplement on bowel movement frequency was defined as a shortened interval between bowel movements; however, other measures of bowel movement frequency or bowel movement probability were also evaluated.

Paying attention to the observation that bowel movement tends to occur with the lapse of time, the bowel movement interval of each individual was modeled by the Weibull distribution. The Weibull distribution is widely used to investigate the effects of drugs on survival time, and it has a shape parameter and a scale parameter [55]. The effect of intake itself was estimated based on a proportional hazards model, assuming that intake influences the scale parameter. The corresponding mathematical formulas are shown below.

$$I_{s,i}∼Weibull\left(k_{s},\lambda_{s,i}\right)$$

$$k_{s}∼HalfNormal\left(0,\sigma_{k}\right)$$

$$\lambda_{s,i}=\left\{\begin{array}{cc} \exp(gut\_state_{s}+\beta_{s,placebo}+\beta_{s,test}) & \left(if\;eating\;Bifidobacterium\right)\\ \exp(gut\_state_{s}+\beta_{s,placebo}) & \left(if\;eating\;placebo\;\sup plement\right)\\ \exp(gut\_state_{s}) & \left(else\right) \end{array}\right)$$

$$gut\_state_{s}∼Normal\left(0,\sigma_{gut\_state}\right)$$

$$\beta_{s,\text{placebo}}∼\text{Normal}\left(0,\sigma_{\text{placebo}}\right)$$

$$\beta_{s,\text{test}}∼\text{Normal}\left(0,\sigma_{\text{test}}\right)$$

s:subjectindex

i:stoolindex

$$I_{s,i}:\text{time interval of}\;i\text{th stool of subject}\;s$$

$$k_{s}:\text{shape parameter of subject}\;s$$

$$\lambda_{s,i}:\text{scale parameter of}\;i\text{th stool of subject}\;s$$

$$\sigma_{k}:\text{scale parameter of shape parameter}\;k$$

$$gut\_state_{s}:\text{personal variable parameter to explain normal gut status}$$

$$\beta_{s,placebo}:personal\;\mathrm{var}iable\;parameter\;to\;\exp lain\;\mathrm{int}ake\;effect\;occur\;in\;both\;Bifidobacterium\;and\;controll\;\sup plement$$

$$\beta_{s,test}:personal\;\mathrm{var}iable\;parameter\;to\;\exp lain\;\mathrm{int}ake\;effect\;occur\;in\;only\;Bifidobacterium\;\sup plement$$

$$\sigma_{gut\_state}:scale\;paramter\;of\;gut\_state$$

$$\sigma_{\mathrm{placebo}}:scale\;paramter\;of\;\beta_{s,placebo}$$

$$\sigma_{\mathrm{test}}:scale\;paramter\;of\;\beta_{s,test}$$

In this model, it is assumed that the effect of each variable at time t occurs as a product of the hazard function in the basis state. The hazard function is a function that expresses the probability of occurrence of an event after time lapse Δt from time t. In this study, it was assumed that the effect of the variable accompanying the bowel movement time, such as the influence of the intake of the test supplement, occurs as a product of the hazard function in the normal state and that the occurrence probability of bowel movement changes. This assumption is equivalent to the assumption that factors other than intake do not influence the hazard function throughout the observation period. This assumption is supported by the empirical observation that the condition in the intestine is stable in a state where food intake is strictly restricted, as it is in the cohort of the present study. Moreover, the parameters in the Weibull distribution in the model and the effect of intake are unknown. Such values were estimated from the observed data. The record of bowel movements acquired in this study did not include the time of each movement, but reported the number of bowel movements per day. Therefore, the interval between successive bowel movements was estimated by dividing 24 h by the frequency. One subject (MO03) consistently had one bowel movement a day throughout the entire observation period of 85 days; thus, the bowel movement interval was always 24 h. Since a variable with zero variance is inappropriate for model estimation, this subject was excluded from subsequent analysis. Furthermore, multiple models were constructed and compared using the Widely Applicable Information Criterion (WAIC)*. WAIC approximates the prediction error (generalization error) for unknown data in the model; WAIC has also been used for a model in which the posterior distribution of parameters cannot be approximated by a normal distribution [56].

Parameters were estimated using a Hamiltonian Monte Carlo method based on the NUTS algorithm with Python and Stan [57]. A generalized linear model of the Weibull distribution using Markov Chain Monte Carlo (MCMC) was applied as described in a previous study [58]. The number of MCMC iterations was 20,000, the number of chains was 8, and the first 1,000 steps in each iteration were discarded as burn-in. In a previous study, MCMC convergence was confirmed by a potential scale reduction factor (PSRF or Rhat) <1.1 for all estimates [59]. Similarly, in the present study, Rhat was calculated based on the variance of multiple MCMC chains and was used for the convergence determination. Initial values were used for the other parameters. The implemented software is available from https://github.com/metagen/EEBIIC.

### 5.10 Other defecation responder estimation models

The defecation dynamics were modeled as defecation frequency and defecation probability. Regarding defecation frequency, it was assumed that the number of defecations per day for each individual followed a Poisson distribution. The Poisson distribution is used when modeling count data and has one ratio parameter. The effect of ingestion was estimated assuming that the ratio parameter changes according to ingestion. For this method, the questionnaire-based defecation frequencies obtained from each subject were used.

Regarding defecation probability, it was assumed that the occurrence of defecation per day of each individual can be modeled by the Bernoulli distribution. The Bernoulli distribution is used in expressing the occurrence probability of alternative choices, such as the side of a coin, and has one probability parameter. The effect of ingestion was estimated assuming that the probability parameter changes through a logistic function according to ingestion. For this method, the defecation frequency data were converted to presence/absence data for defecation each day, with 1 recorded if a bowel movement occurred and 0 otherwise.

### 5.11 Prediction of responders by machine learning and feature analysis of responders using a statistical analysis

We used the relative abundance of bacterial genera and the fecal metabolite contents before the test intervention (T1) in all subjects except MO03 in the 1st cohort as feature values. MO03 was excluded from this analysis because the bowel movements of this subject always occurred once a day throughout the whole observation period (85 days), which was not suitable for determining responders by the Bayesian statistical model. We used 41 samples when analyzing with only the microbiome data, and when combined with metabolome data, we used 19 samples. Of all feature values, those of the relative abundance or the fecal content exceeding a certain threshold were selected. For the threshold value, a grid search was conducted to search for the one with the maximum predicted AUROC. Subsequently, each feature value was standardized by centering to a mean of 0 and dividing by the standard deviation (z-score) of each feature, considering the following potential problems: 1) it is possible that a feature value with a large range of possible values will have a large influence on the prediction because the range of values that can be taken with the microbial relative abundance and metabolite contents are different; and 2) the feature importance of the microbiome and metabolome cannot be directly compared. Nineteen subjects were classified into two groups, responders (SRs and WRs) and NRs. To identify metabologenomic markers that can distinguish responders from NRs, we constructed classification models based on metabologenomic data using LASSO regression and logistic regression algorithms. The model was validated by 7-fold stratified cross-validation testing (we resampled dataset partitions 10 times). A LASSO regression model was used for feature selection from the training dataset, which was fitted to the mean value of the increase in bowel movement frequency with the test supplement for each individual estimated by a statistical model. Only the features with nonzero coefficients were extracted. Regarding the LASSO regression hyperparameters, a grid search was performed to search for the parameter set with the maximum predicted AUROC. Test data were predicted by a logistic regression model learned from feature-selected training data. For a hyperparameter in the logistic regression, we used the default parameters in sklearn.linear_model.LogisticRegression (version 0.19.1).

The hyperparameter and accuracy are described in Table 1. In the analysis of responder characteristics, similar to machine learning, the values from 41 samples collected just before intake were used, and we also used the rate of change after the test food intake (defined as responder scores) in the analysis. Responder scores were compared with values obtained after test food intake and placebo intake, after considering the baseline values. The responder scores are defined using the following formulas:

1weekResponderScore=((T2-T1)-(C2-C1))/Average(C1,T1)

2weekResponderScore=((T3-T1)-(C3-C1))/Average(C1,T1)

In statistical analysis, a two-sided Jonckheere-Terpstra trend test was applied to the NR, WR, and SR groups, and those with *p*-values less than 0.05 were extracted.

### 5.12 Clinical trial of the second cohort

An analysis of the second cohort was performed to confirm the findings of the main trial. In the second cohort, a three-way randomized double-blind controlled crossover trial with healthy subjects was performed. The inclusion and exclusion criteria were basically the same as those in the main trial, but the frequency of defecation in the inclusion criteria was 3–5 times per week in second cohort. The responder estimation described in “5.9. Bowel responder definition based on the Bayesian Weibull regression model” was performed for the two periods in which placebo/*B. longum* BB536 capsules were used. In addition, the microbiome was analyzed from the fecal samples collected at the time point just before the use of the *B. longum* BB 536 capsule.

## Author Contributions

S.F. and T.Y. conceived and designed the project. M.K., T.Y., and S.F. designed the clinical trials. M.K. prepared the test supplement used for intervention. S.M., C.I., and Y.I. extracted DNA from fecal samples. C.I. and Y.I. performed the microbiome analysis. S.M., C.I., and Y.I. performed the CE-TOFMS-based metabolome analysis. Yuya N. conducted the statistical analysis. Yuya N. and Shinya S. performed all analyses related to metabologenomic data. Yuya N. and Yuichiro N. predicted responders from metabologenomic data using machine learning. Y.M. performed the PCR amplification of the 16S rRNA gene region of *Bifidobacterium*. Yuichiro N., N.W., K.H., and J.U. contributed to the study design. Yuya N., Shinya S. and Yuichiro N. wrote the initial draft of the manuscript. S.F. and T.Y. supervised the project and wrote the paper. All authors reviewed and approved the final version of the paper.

## Funding statement

None declared.

## Data availability statement

The raw sequencing dataset reported in this paper is available in the DDBJ Sequence Read Archive (DRA006874).

## CRediT authorship contribution statement

**Yuya Nakamura:** Writing – original draft, Formal analysis, Methodology. **Shinya Suzuki:** Writing – original draft, Methodology. **Shinnosuke Murakami:** Investigation. **Yuichiro Nishimoto:** Writing – original draft, Methodology. **Koichi Higashi:** Investigation. **Naoki Watarai:** Investigation. **Junpei Umetsu:** Investigation. **Chiharu Ishii:** Investigation. **Yutaro Ito:** Investigation. **Yuka Mori:** Investigation. **Mamiko Kohno:** Resources. **Takuji Yamada:** Conceptualization, Supervision, Writing – review & editing. **Shinji Fukuda:** Conceptualization, Supervision, Writing – review & editing.

## Declaration of Competing Interest

The authors declare that they have no known competing financial interests or personal relationships that could have appeared to influence the work reported in this paper.
