# Supplementary material for: Integrated gut microbiome and metabolome analyses identified fecal biomarkers for bowel movement regulation by Bifidobacterium longum BB536 supplementation: A RCT
Source: Comput Struct Biotechnol J. 2022 Oct 25;20:5847–58. doi: 10.1016/j.csbj.2022.10.026 (PMC9636538; doi:10.1016/j.csbj.2022.10.026)
Supplement: Supplementary data 1 [file mmc1.docx]

Figure S1. MDS using beta diversity. A: Scatter plot of MDS-derived beta diversity (Spearman correlation coefficient distance) calculated from the microbiome composition. B: Scatter plot of MDS-derived beta diversity (Spearman correlation coefficient distance) calculated from the metabolite composition. Different colors denote different time points.

Figure S2. Verification of the carry-over effect:

Spearman coefficient distances between the microbiome (A) or metabolome (B) between Period 1 and Period 2 of each test subject just before supplementation were calculated and shown in groups. No significant difference was detected in either microbiome or metabolome, and no carry-over effect was observed (n. s.: not significant).


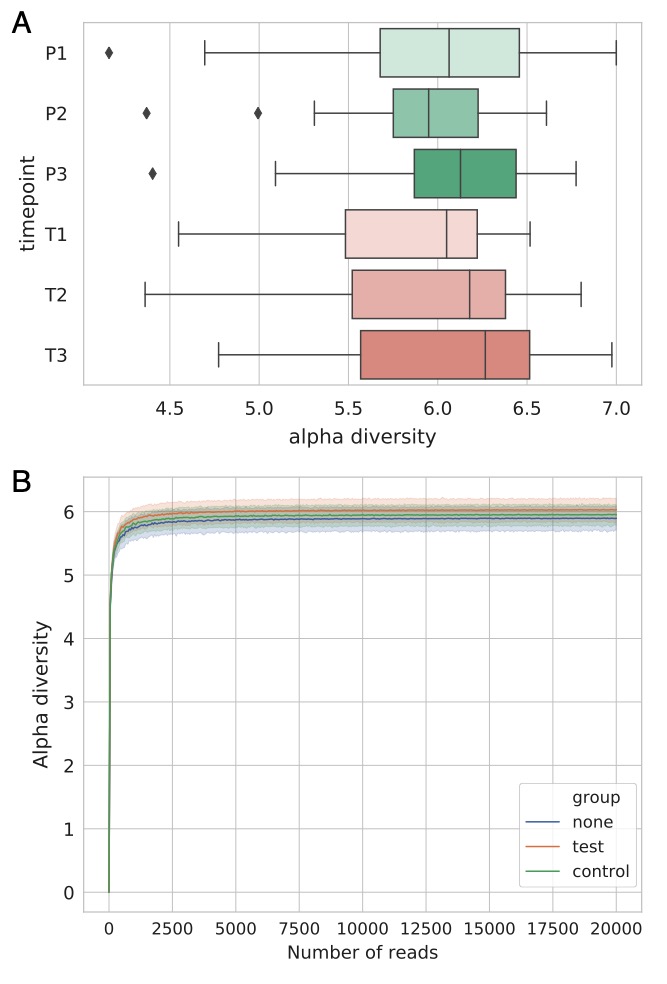


Figure S3. Microbial alpha diversity. A: Boxplot of alpha diversity (Shannon diversity index) calculated from the microbiome composition. B: Rarefaction curve of alpha diversity.


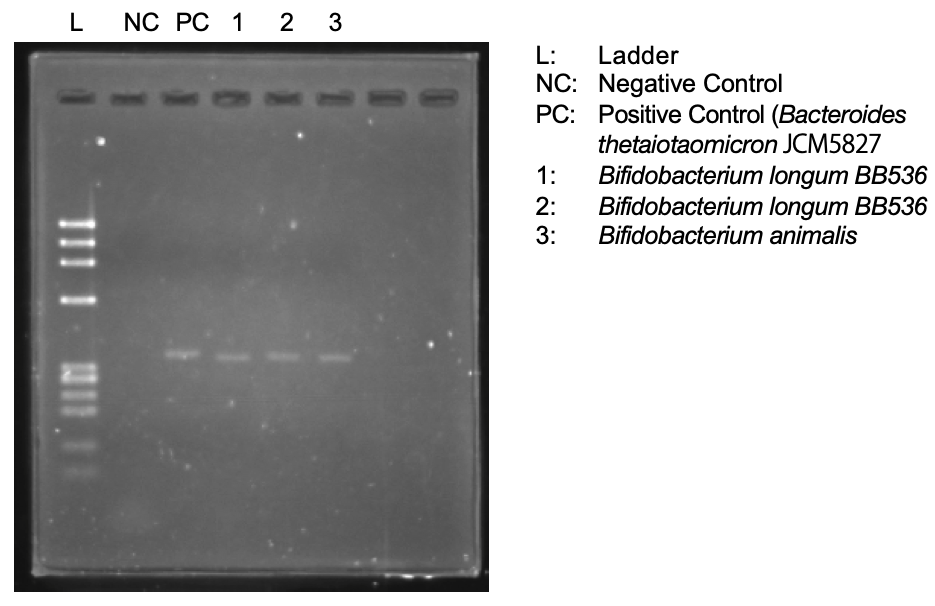


Figure S4. Confirmation of whether *Bifidobacterium longum* BB536 is increased by universal primers. From left, L; ladder, NC; negative control, PC; positive control (*Bacteroides thetaiotaomicron* JCM5827), 1, 2; *Bifidobacterium longum* BB536 and 3; *Bifidobacterium animalis.*
